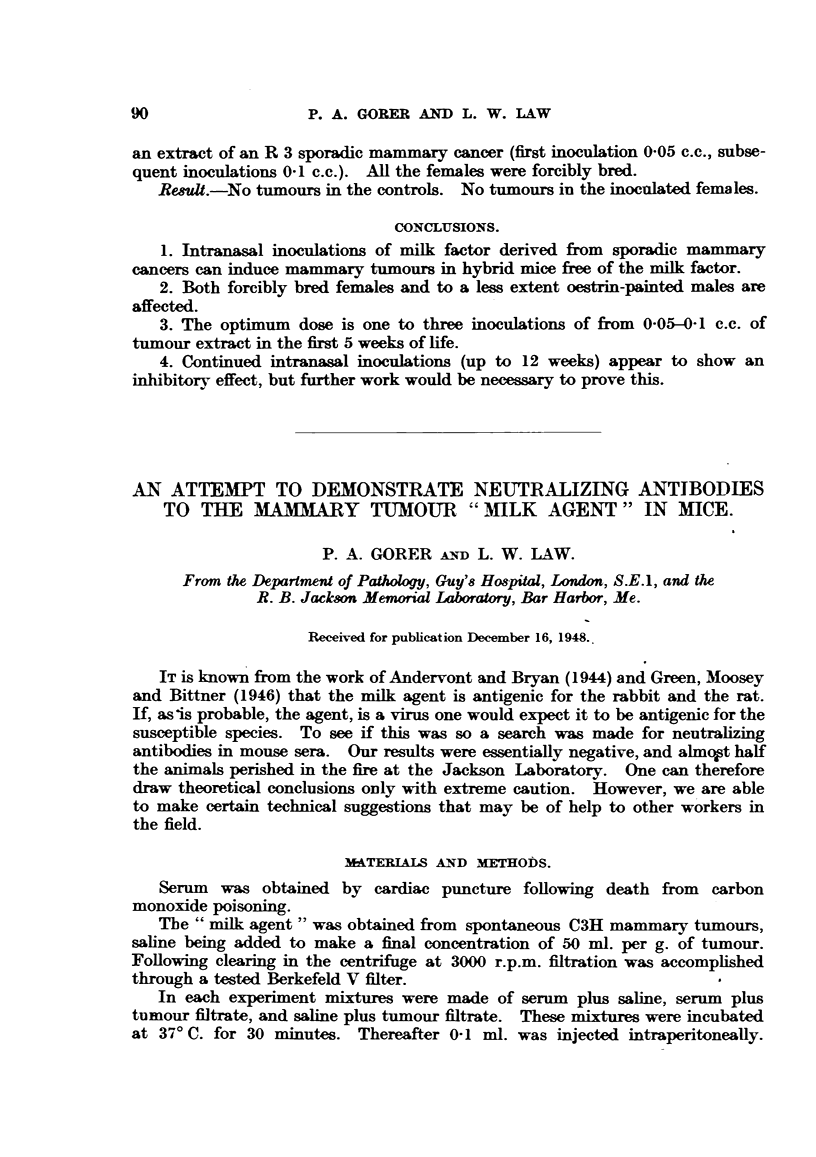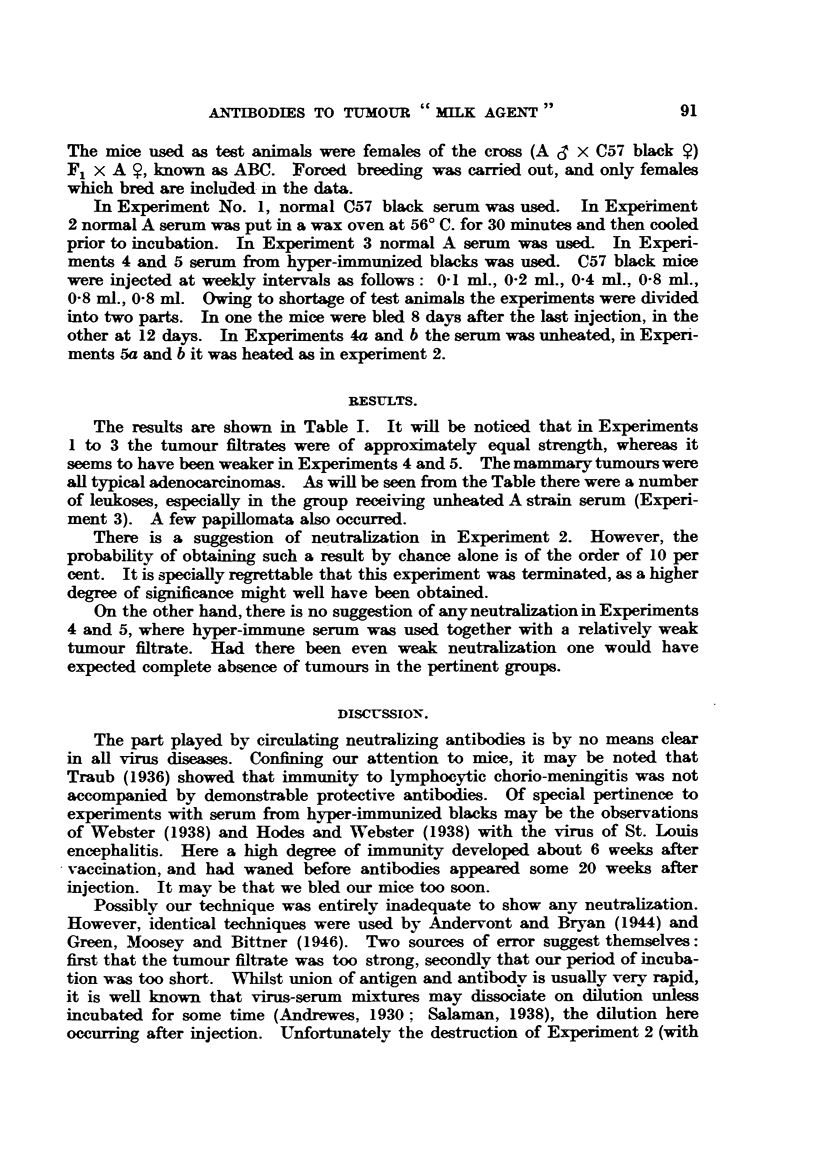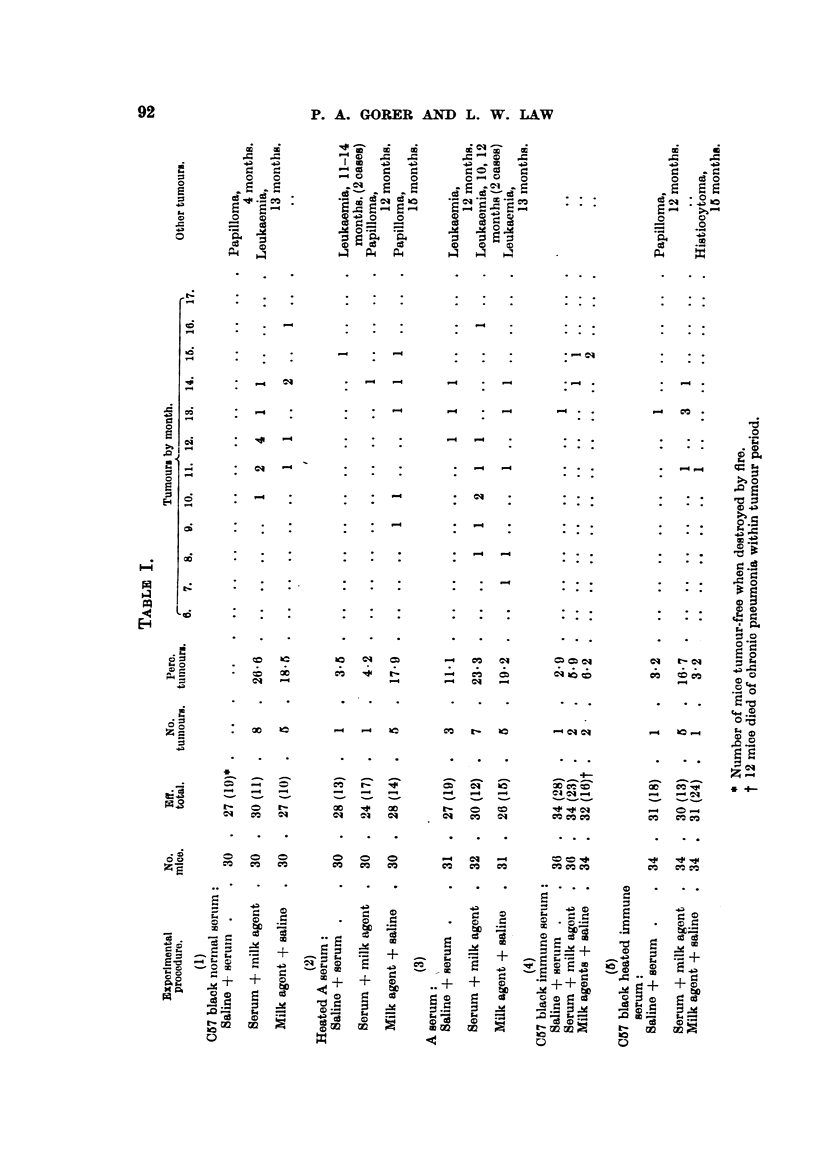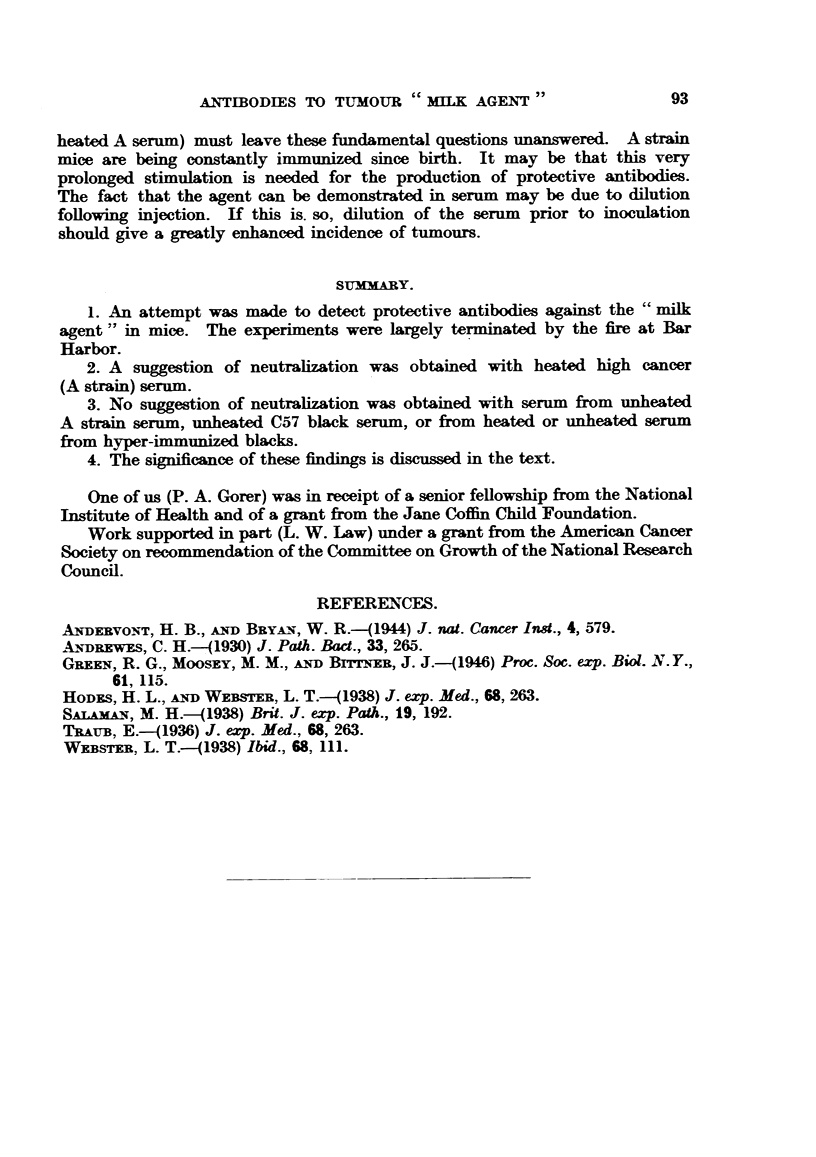# An Attempt to Demonstrate Neutralizing Antibodies to the Mammary Tumour “Milk Agent” in Mice

**DOI:** 10.1038/bjc.1949.10

**Published:** 1949-03

**Authors:** P. A. Gorer, L. W. Law


### AN ATTEMPT TO DEMONSTRATE NEUTRALIZING ANTIBODIES TO THE MAMMARY TUMOUR "MILK AGENT" IN MICE.

P. A. GORER AND L. W. LAW.

*From the Department of Pathology, Guy's Hospital, London, S.E.*1, *and the R. B. Jackson Memorial Laboratory, Bar Harbor, Me.*

Received for publication December 16, 1948.

IT is known from the work of Andervont and Bryan (1944) and Green, Moosey and Bittner (1946) that the milk agent is antigenic for the rabbit and the rat. If, as is probable, the agent, is a virus one would expect it to be antigenic for the susceptible species. To see if this was so a search was made for neutralizing antibodies in mouse sera. Our results were essentially negative, and almost half the animals perished in the fire at the Jackson Laboratory. One can therefore draw theoretical conclusions only with extreme caution. However, we are able to make certain technical suggestions that may be of help to other workers in the field.

MATERIALS AND METHODS.

Serum was obtained by cardiac puncture following death from carbon monoxide poisoning.

The "milk agent" was obtained from spontaneous C3H mammary tumours, saline being added to make a final concentration of 50 ml. per g. of tumour. Following clearing in the centrifuge at 3000 r.p.m. filtration was accomplished through a tested Berkefeld V filter.

In each experiment mixtures were made of serum plus saline, serum plus tumour filtrate, and saline plus tumour filtrate. These mixtures were incubated at 37° C. for 30 minutes. Thereafter 0·1 ml. was injected intraperitoneally.

The mice used as test animals were females of the cross (A ♂ × C57 black ♀) $F_1$ × A ♀, known as ABC. Forced breeding was carried out, and only females which bred are included in the data.

In Experiment No. 1, normal C57 black serum was used. In Experiment 2 normal A serum was put in a wax oven at 56° C. for 30 minutes and then cooled prior to incubation. In Experiment 3 normal A serum was used. In Experiments 4 and 5 serum from hyper-immunized blacks was used. C57 black mice were injected at weekly intervals as follows: 0·1 ml., 0·2 ml., 0·4 ml., 0·8 ml., 0·8 ml., 0·8 ml. Owing to shortage of test animals the experiments were divided into two parts. In one the mice were bled 8 days after the last injection, in the other at 12 days. In Experiments 4*a* and *b* the serum was unheated, in Experiments 5*a* and *b* it was heated as in experiment 2.

### RESULTS.

The results are shown in Table I. It will be noticed that in Experiments 1 to 3 the tumour filtrates were of approximately equal strength, whereas it seems to have been weaker in Experiments 4 and 5. The mammary tumours were all typical adenocarcinomas. As will be seen from the Table there were a number of leukoses, especially in the group receiving unheated A strain serum (Experiment 3). A few papillomata also occurred.

There is a suggestion of neutralization in Experiment 2. However, the probability of obtaining such a result by chance alone is of the order of 10 per cent. It is specially regrettable that this experiment was terminated, as a higher degree of significance might well have been obtained.

On the other hand, there is no suggestion of any neutralization in Experiments 4 and 5, where hyper-immune serum was used together with a relatively weak tumour filtrate. Had there been even weak neutralization one would have expected complete absence of tumours in the pertinent groups.

### DISCUSSION.

The part played by circulating neutralizing antibodies is by no means clear in all virus diseases. Confining our attention to mice, it may be noted that Traub (1936) showed that immunity to lymphocytic chorio-meningitis was not accompanied by demonstrable protective antibodies. Of special pertinence to experiments with serum from hyper-immunized blacks may be the observations of Webster (1938) and Hodes and Webster (1938) with the virus of St. Louis encephalitis. Here a high degree of immunity developed about 6 weeks after vaccination, and had waned before antibodies appeared some 20 weeks after injection. It may be that we bled our mice too soon.

Possibly our technique was entirely inadequate to show any neutralization. However, identical techniques were used by Andervont and Bryan (1944) and Green, Moosey and Bittner (1946). Two sources of error suggest themselves: first that the tumour filtrate was too strong, secondly that our period of incubation was too short. Whilst union of antigen and antibody is usually very rapid, it is well known that virus-serum mixtures may dissociate on dilution unless incubated for some time (Andrewes, 1930; Salaman, 1938), the dilution here occurring after injection. Unfortunately the destruction of Experiment 2 (with heated A serum) must leave these fundamental questions unanswered. A strain mice are being constantly immunized since birth. It may be that this very prolonged stimulation is needed for the production of protective antibodies. The fact that the agent can be demonstrated in serum may be due to dilution following injection. If this is. so, dilution of the serum prior to inoculation should give a greatly enhanced incidence of tumours.

SUMMARY.

1. An attempt was made to detect protective antibodies against the " milk agent " in mice. The experiments were largely terminated by the fire at Bar Harbor.

2. A suggestion of neutralization was obtained with heated high cancer (A strain) serum.

3. No suggestion of neutralization was obtained with serum from unheated A strain serum, unheated C57 black serum, or from heated or unheated serum from hyper-immunized blacks.

4. The significance of these findings is discussed in the text.

One of us (P. A. Gorer) was in receipt of a senior fellowship from the National Institute of Health and of a grant from the Jane Coffin Child Foundation.

Work supported in part (L. W. Law) under a grant from the American Cancer Society on recommendation of the Committee on Growth of the National Research Council.

REFERENCES.

Andervont, H. B., and Bryan, W. R.—(1944) *J. nat. Cancer Inst.*, **4**, 579.

Andrewes, C. H.—(1930) *J. Path. Bact.*, **33**, 265.

Green, R. G., Moosey, M. M., and Bittner, J. J.—(1946) *Proc. Soc. exp. Biol. N.Y.*, **61**, 115.

Hodes, H. L., and Webster, L. T.—(1938) *J. exp. Med.*, **68**, 263.

Salaman, M. H.—(1938) *Brit. J. exp. Path.*, **19**, 192.

Traub, E.—(1936) *J. exp. Med.*, **68**, 263.

Webster, L. T.—(1938) *Ibid.*, **68**, 111.